# iSUMOK-PseAAC: prediction of lysine sumoylation sites using statistical moments and Chou’s PseAAC

**DOI:** 10.7717/peerj.11581

**Published:** 2021-08-04

**Authors:** Yaser Daanial Khan, Nabeel Sabir Khan, Sheraz Naseer, Ahmad Hassan Butt

**Affiliations:** Department of Computer Science, School of Systems and Technology, University of Management and Technology, Lahore, Punjab, Pakistan

**Keywords:** Post translation modification, Lysine Sumoylation, Computational protemics, Neural networks, Statistical moments, Hahn moments

## Abstract

Sumoylation is the post-translational modification that is involved in the adaption of the cells and the functional properties of a large number of proteins. Sumoylation has key importance in subcellular concentration, transcriptional synchronization, chromatin remodeling, response to stress, and regulation of mitosis. Sumoylation is associated with developmental defects in many human diseases such as cancer, Huntington’s, Alzheimer’s, Parkinson’s, Spin cerebellar ataxia 1, and amyotrophic lateral sclerosis. The covalent bonding of Sumoylation is essential to inheriting part of the operative characteristics of some other proteins. For that reason, the prediction of the Sumoylation site has significance in the scientific community. A novel and efficient technique is proposed to predict the Sumoylation sites in proteins by incorporating Chou’s Pseudo Amino Acid Composition (PseAAC) with statistical moments-based features. The outcomes from the proposed system using 10 fold cross-validation testing are 94.51%, 94.24%, 94.79% and 0.8903% accuracy, sensitivity, specificity and MCC, respectively. The performance of the proposed system is so far the best in comparison to the other state-of-the-art methods. The codes for the current study are available on the GitHub repository using the link: https://github.com/csbioinfopk/iSumoK-PseAAC.

## Introduction

Post-translational modifications are the chemical changes that occur during the structural and functional organization of a protein after the protein is synthesized by the translational process. These chemical reactions or changes that take place at certain amino acid residue after the translational process are known as post-translational modifications (PTMs). The post-translational modifications can be covalent or enzymatic. These modifications involve phosphorylation, glycosylation, ubiquitination, nitrosylation, methylation, acetylation, lipidation, and proteolysis have impacted all the details of cellular biology and pathogenesis. The post-translational modification also has involvement in protein for various functions with a minimal number of genes and can influence protein actions such as gene expressions. PTM modulates the cellular behavior and relates to any change in the amino acid chains of the protein after its alteration. They are moderated by enzymatic activity and can be reversed according to its change using enzymatic actions (Lu et al., 2010; Zhao et al., 2014; Beauclair et al., 2015).

Small ubiquitin-like modifier (SUMO) is like a group of tiny proteins that may be covalently similar to other proteins in the cells and alter their functions. Sumoylation is a post-translational modification that performs various cellular functions such as nuclear-cytosolic transport, transcriptional ordinance, protein reliability, and development throughout the cellular cycle. SUMO is a reversible PTM in which small ubiquitin proteins are covalently bound to a lysine residue in a process same as ubiquitylation. SUMO is constructed after the binding of the final four amino acids having C terminals and is responsible for an iso-peptide bond among the C terminal glycine residue of SUMO and lysine (Green, Dmochowski & Golshani, 2006; Geiss-Friedlander & Melchior, 2007).

SUMO is mainly contained in a chain of 97 amino acids named as Smt3p, Pmt2p, PIC-1, GMP-1, Ubl1, and Sentrin. Modification of cellular proteins such as ubiquitin-like proteins, SUMO is necessary for many eukaryotic cellular procedures and cell cycle development in yeast. SUMO also effects plants and vertebrates. Plants have eight SUMO isoforms. In mammals, SUMO has four isoforms: SUMO1, SUMO2, SUMO3, and SUMO4. Lymph nodes, kidneys, and spleen in mammals are identified using SUMO4. Sumoylation is helpful in many biomechanical processes such as gene expression, DNA repair, chromosome recombination, and cell signaling (Müller et al., 2001; Hay, 2005; Geiss-Friedlander & Melchior, 2007; Ijaz, 2013). Many different types of diseases like cancer (Seeler et al., 2007), inherited heart defects (Wang et al., 2011), diabetes (Zhao, 2007), and neurodegenerative diseases (Lee et al., 2013), *etc*., are directly linked to Sumoylation synchronization and modulation.

SUMO modified proteins have peptide concord motifs Ψ, K, x, and D/E. Here ‘Ψ’ is the hydrophobic amino acid, ‘k’ is the lysine residue, ‘x’ is the amino acid and ‘E’ is the glutamic acid. The consensus motifs were examined with a percentage of 23% in the preliminary study. Furthermore, in recent studies (Rodriguez, Dargemont & Hay, 2001; Sampson, Wang & Matunis, 2001; Xue et al., 2006) ψ-K-x-E\D was the consensus motif and reveals that 26% of SUMO sites did not accompany the consensus motifs. In short from the current study on Sumoylation it was observed that 40% of sumoylation sites did not have the consensus motifs (Zhao et al., 2014), and the remaining used the consensus motifs. This lack of information has led to insufficient knowledge for Sumoylation.

Lysine is a post-translational modification residue. It is the most used amino acid of PTM having Sumoylation and also narrates for various sumoylation sites. Lysine sumoylation is the reversible most modulated PTM. It lies *via* the covalent link of a small ubiquitin-like modifier (SUMO) and is moderated by the generation of enzymes E1 link enzymes E2 and ligase E3. Lysine residue endures many PTM as there must be synchronization among them (Müller et al., 2001).

In past, biologists were expected to perform the traditional experiments with the utilization of costly equipment in order to identify the post-translation modification of a protein. In recent years, the improvement of bioinformatics permitted scientific community to solve complex protein problems by the combination of informatics, mathematics, and statistics. Due to the reversible behavior of sumoylation, it was observed that the different procedures or methods used for the deficiency of sumoylation site modification must be essential for the site prediction process.

Green, Dmochowski & Golshani (2006) proposed parallel cascade identification method for the prediction of Sumoylation sites. The main purpose behind this method was to originate PCI based proteomics tools for the prediction of protein structure and function. Xue et al. (2006) introduced a novel computational method SUMOsp for the prediction of Sumoylation sites. SUMOsp was based on manually curated datasets using the integration of two methods, GPS and MotifX. SUMOsp used large datasets and is considered a vigorous tool for *in vivo* and *in vitro* sumoylation site prediction. Zhao et al. (2014) proposed a GPS-SUMO approach used for the identification of sumoylation sites and sumo relating motifs (SIM) in proteins and for examining the association among the sumoylation sites and SUMO relevant techniques. A webserver for GPS-SUMO is also available for the research community to further utilize the dataset and methods. Beauclair et al. (2015) proposed a scoring system based on position frequency matrix derived from the alignment of experimental sumoylation sites. The specified tool, JASSA revealed high gains in proportion of implementation. Therefore JASSA was considered as a costly tool for evaluating the ideal sites and providing useful information about Sumoylation in cellular biology. Chang et al. (2018) proposed SUMOgo using Random Forests, motif screening, and feature selections based on variant in combinations to originate a Sumoylation site prediction. They used sequence-based binary encoding, encoded chemical attributes, and encoded secondary structure details. They enhanced the prediction performance and obtained the MCC 0.51 in comparison to the other state-of-the-art methods. Ijaz (2013) also developed a prediction method that showed improvements in the prediction process of Sumoylation site and its significance. In recent past, López et al. (2020) implemented a computational prediction method using the Adaboost classifier. They utilized the sine and cosine of backbone torsion angles and the accessible surface area. To overcome the balancing issues in their training matrix, they applied NearMiss method with undersampling the majority class. C-iSUMO was effective in use of circular functions. C-iSUMO was effective in prediction of Sumoylation sites as compared to the other state of art systems from the past and achieved 74.6% accuracy with 0.494 MCC.

**Figure 1:**
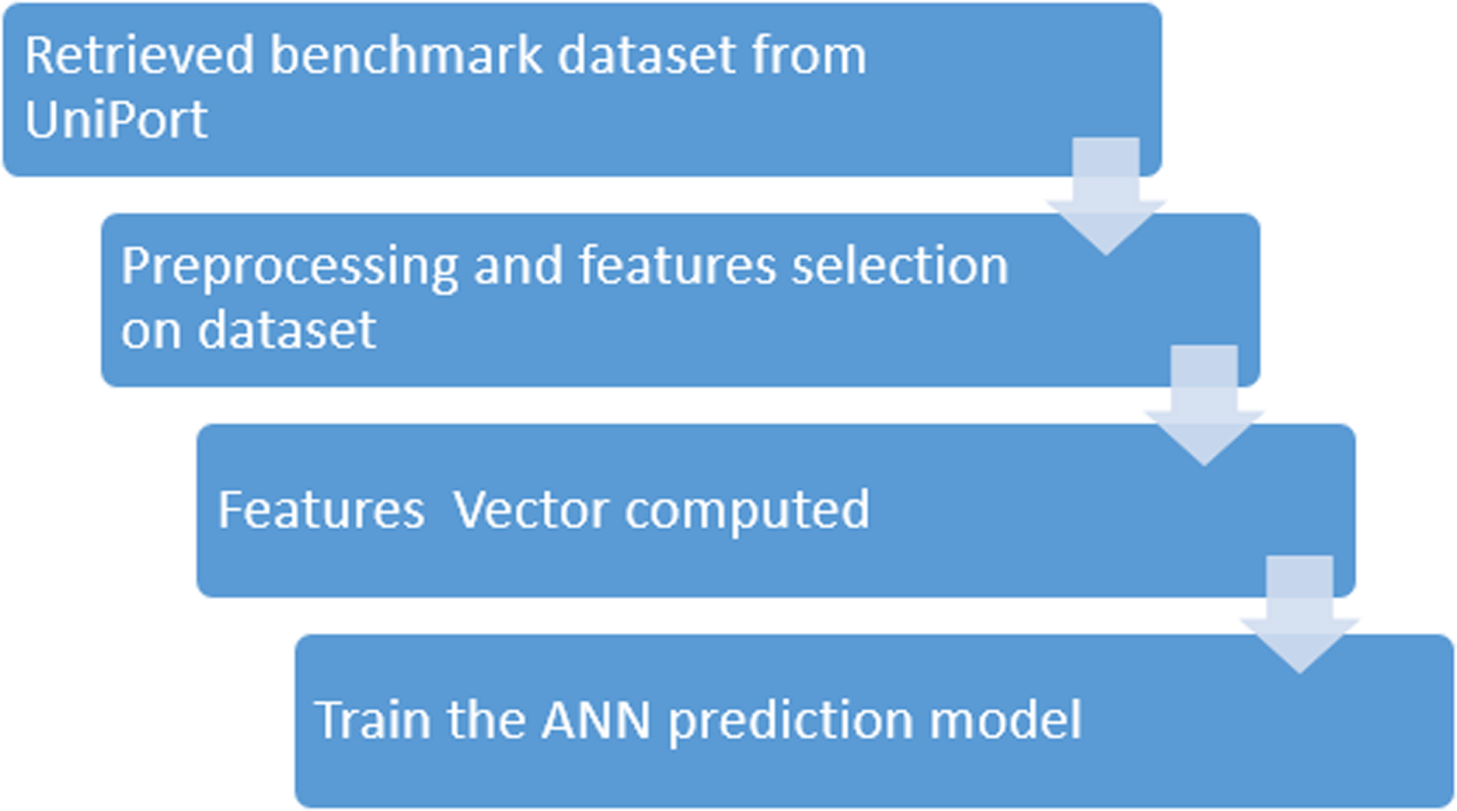
Flowchart of the proposed methodology.

In this study, we propose a novel method iSUMOk-PseAAC, to predict the Sumoylation sites using relative position based features by integrating the Chou’s Pseudo Amino Acid Composition (PseAAC) (Chou, 2001a). The results from the proposed system were compared with the other state of art prediction methods SUMOgo (Chang et al., 2018), GPS-SUMO (Zhao et al., 2014), SUMOsp2.0 (Xue et al., 2006), JASSA (Beauclair et al., 2015), and PCI-SUMO (Green, Dmochowski & Golshani, 2006). The proposed method was implemented using the Chou’s five-step rule (Chou, 2011). This method has been utilized by various studies (Cheng, Xiao & Chou, 2017a, 2017b, 2017c, 2018a, 2018b; Cai et al., 2018; Chen et al., 2018a, 2018b) which follow these five steps: (i) Benchmark dataset construction, (ii) Formulation of Samples, (iii) Operational Algorithm, (iv) Tests using cross-validations, and (v) Implementation of a webserver. These steps are discussed in detail in further sections. The framework used in the proposed system used Chou’s five-step rule. This approach is shown in Fig. 1.

## Materials and Methods

### Benchmark dataset

In many studies, Chou’s peptide formulation approach was used for the simplification of the dataset (Chou, 2001a). Universal resource of protein (UniProtKB) is a freely available as a central repository of protein sequences information. The benchmark dataset was constructed using the UniProtKB. The protein sequences with PTM processing annotations and features were used for the dataset construction of Sumoylation sites. A term named glycl lysine was used for Sumoylation domain to obtain the sequences of SUMO proteins. Only those sites were retrieved and included in datasets which were reviewed and annotated with experimental evidences. There were many redundant sequences or highly similar sequences that existed in the newly constructed dataset. For this purpose, those sequences were removed with the help of a tool CD-HIT (Fu et al., 2012). The similarity threshold value for removing redundant and ambiguous sequences was kept at 60%. Finally, the benchmark dataset was obtained after removing the similar sequences. The negative datasets were also obtained from the UniProtKB using the complement sequences of the searching criteria used for positive Sumoylation sites. The benchmark dataset consists of 4,987 positive and 5,000 negative samples. This dataset was divided into training and testing dataset using the 70:30 ratio. For training data, 3,487 positive and 3,500 negative random samples were selected. Furthermore, an independent testing dataset was constructed from the leftover samples after constructed the training dataset and 3,000 independent test samples were left in which 1,500 are positive and 1,500 are negative samples. In prediction models based on statistical analysis, construction of training and testing datasets is crucial. The benchmark datasets can be accordingly reduced to Eq. (1):

(1) $$K=K^{+}\cup K^{-}$$

**Figure 2:**
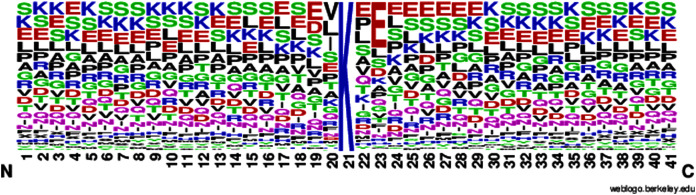
Sumoylation sites.

**Figure 3:**
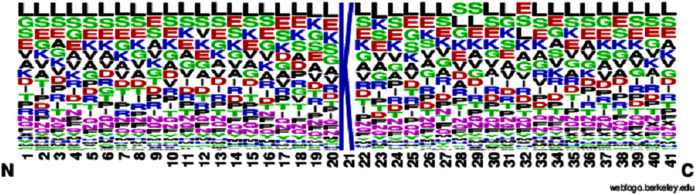
Non-sumoylation sites.

According to Eq. (1), here U is denoted as the union operator. K^+^ represents the 4,987 positive samples and K^−^ represents the 5,000 negative samples. The total sum K consists of 9,987 sample sequences which are provided in the Supplemental Information S1 for the convenience of the readers. The independent dataset is provided in the Supplemental Information S2. For the graphical representation of lysine amino acids, we analyzed the sequence samples by using the development of Web Logo (Chou, 2001a, 2011) (see Figs. 2 and 3). In this logo, the symbol ‘X’ was attached as the dummy code to keep the same length of all samples.

### Sample formulation

As the biological sequences are increasing in biological databanks at exponential rate, the position of discrete models or vectors from the sequences of biological data has been an issue in many bioinformatics based research methods and its attributes for target analysis. There are many algorithms as discussed in the review study (Chou, 2011) for the formulation of sequence in vector form, but the machine learning algorithms are among the top-rated algorithms such as Random Forest (RF) algorithm (Lin et al., 2011; Jia et al., 2016), Covariance Discriminant (CD) (Chou & Elrod, 2002; Lin et al., 2012), *etc*. The pseudo amino acid composition (PseAAC) (Chou, 2001b) was proposed to overpower the sequence pattern-related information of protein which might be a loss if they are represented in the form of vector or discrete model. Chou’s PseAAC has been utilized for many computational proteomics problems (Butt et al., 2016; Butt, Rasool & Khan, 2017, 2018, 2019; Sabooh et al., 2018; Sankari & Manimegalai, 2018; Srivastava, Kumar & Kumar, 2018; Zhang & Liang, 2018; Zhao et al., 2018; Butt & Khan, 2020a, 2020b; Chou, 2001c). Because of its prevalence and significance in computational proteomics, there are three webservers for the facilitation of the research community called PseAAC-General (Du, Gu & Jiao, 2014), PseAAC-Builder (Du et al., 2012), and propy (Cao, Xu & Liang, 2013). The previous two were used for Chou’s PseAAC by many researchers [68] but the initial one was used for functional domains, mods, gene ontology, and sequential evaluation of Chou’s general PseAAC (Chou, 2011). After using PseAAC for many protein prediction problems, PseKNC was proposed for the computation of features vectors using DNA/RNA sequences (Liu et al., 2015a; Chen et al., 2016a; Feng et al., 2017). Nowadays, most competent and effective webserver named ‘Pse in One’ (Liu et al., 2015b) and ‘Pse in One2.0’ (Liu, Wu & Chou, 2017) was evolved for feature vector design from protein sequence, peptide sequence and DNA/RNA sequence. By applying the Chou’s general PseAAC (Chou, 2011), protein site sequence ‘**S**’ can be represented as Eq. (2):

(2) $$S=N_{1}N_{2}N_{3}N_{4}N_{5}N_{6}\ldots \;N_{P}$$

Here $N_{1}$ in protein sample ‘S’ represents the first residue, $N_{2}$ as the second amino acid residue, and similarly, $N_{P}$, as the last amino acid residue. *P* is the total length of the protein site sequence or the total number amino acids in a sequence. The following discrete model based on amino acid composition is used to represent protein **S** in Eq. (3):

(3) $$S=[F_{1\;}\;F_{2}\;\;F_{3}\ldots \;F_{20}{]}^{T}$$

The extraction of useful features is very important from the relevant protein/peptide sequences which are explained in Eq. (3). Where *F_X_* (X = 1, 2, 3, …, 20) are the useful amino acid features and symbol *T* is the transpose of a site sequence of the protein **S**. Each sample site in dataset was represented as a peptide segment of length 41 with 20 amino acid residues upstream and 20 residues downstream of the amino acid residue ‘K’ lysine. The following Eq. (4) represents the modified sample sequence from Eq. (2):

(4) $$S=N_{1},N_{2},N_{3},\ldots N_{19},N_{20},N_{21}\ldots \;N_{22},\;N_{23},N_{24},\;\ldots \;N_{41}$$

Here $N_{21}=K$ which represents the target lysine residue and $N_{P}\left(P=1,2,3,\;\ldots ,41;P\neq 21\right)$ can be any other amino acid or virtual X code. A virtual amino acid residue ‘X’ fills the positions where no sufficient residues exist to make sure that the length of the peptide segment remains unified. From now onwards, the numerical codes of amino acid used as per order of their name as alphabetical order according to their first letter, 1, 2, 3 …… 20 for 1 to all 20, one per amino acid and 21 for X.

#### Site vicinity vector

In the polypeptide chain, the chance of PTM is much higher. There are multiple reasons for that modification such as lysine ubiquitination sites, methylation sites, sumoylation sites, and numerous other lysine PTM sites. Observing the potential site along with neighboring residues where PTM is also an important aspect (Lo & Don, 1989). A small sequence that contains the potential PTM residue site from the main sequence is called Site Vicinity Vector (SVV).

Let Ωi be the variable that shows the possible PTM site containing neighboring residues in the main sequence are given below in Eq. (5)

(5) $$S=[\Omega_{1}\ldots \Omega_{i-2},\Omega_{i-1},\Omega_{i},\Omega_{i+1},\Omega_{i+2},\ldots \Omega_{n}]$$

The SVV can be defined as a subsequence of the primary sequence as given below as Eq. (6):

(6) $$S=[\Omega_{i-j}\ldots \Omega_{i-2},\Omega_{i-1},\Omega_{i},\Omega_{i+1},\Omega_{i+2},\ldots \Omega_{i+j}]$$

The symbol i holds the minimum value of 20. In subsequence of protein where every residue symbolizes a specific amino acid out of known 20 amino acids. In SVV to assigned the unique numerical code to each residue position from the range of 1–20 of amino acid and one value 21 for the virtual amino acid ‘*X*’. The value of i and j is the size of the window, which is chosen after extensively probing different values from a given range (10–100). The values that outcome best performance in results are selected and used accordingly.

#### Statistical moments

In most problems of pattern recognition, the statistical moment based features have been widely used for the qualitative measures of benchmarks datasets. Due to sensitive information of the sequence of amino acid residue in the benchmark dataset, a statistical approach was applied to manage the order of sequence samples of protein. The collection of numerous types of information was derived from a variety of statistical moments from the dataset of protein samples, to use the evaluation of data size and some other orientation and eccentricity. Various kinds of moments were used by the mathematicians and statisticians (Khan, Ahmad & Anwar, 2012; Khan, Ahmed & Khan, 2014) and these moments are depended on the distribution functions and polynomials.

Moments are a collection of statistical parameters used to understand the characteristics of a function and to capture its significance features; therefore Hahn, Raw and Central moments were calculated for the iSUMOk-PseAAC prediction model. Hahn moments are scale and position variant (Khan, 2014a) and calculated using the Hahn polynomials. Raw moments are also scaled and position variant calculated using the probability distribution of the dataset. Raw moments are very helpful for the calculation of asymmetry of samples of proteins, variance, and mean of benchmark dataset. Furthermore, central moments were also calculated which are scale variant and vicinity invariant (Butt et al., 2016; Butt, Rasool & Khan, 2017) and based on a centroid. It also has the property for the computation of variance, asymmetry, and mean of benchmark dataset.

The proposed method used a two-dimensional matrix Ṕ having (n × n) dimensions that contain all the samples of proteins of the dataset. In this study, each method characterizes a benchmark dataset that represents quantified values and by passes the scale variant moments (Khan et al., 2014b).

(7) $$P=\;\left[\begin{array}{c} \begin{array}{cc} R_{1\to 1} & R_{1\to 2}\\ R_{2\to 1} & R_{2\to 2} \end{array}\\ \begin{array}{cc} \vdots & \vdots \\ R_{x\to 1} & R_{x\to 2} \end{array}\\ \begin{array}{cc} \vdots & \vdots \\ R_{n\to 1} & R_{n\to 2} \end{array} \end{array}\begin{array}{c} \begin{array}{cc} \cdots & R_{1\to y}\\ \cdots & R_{2\to y} \end{array}\\ \begin{array}{cc} \cdots & \vdots \\ \cdots & R_{x\to y} \end{array}\\ \begin{array}{cc} \cdots & \vdots \\ \cdots & R_{n\to y} \end{array} \end{array}\begin{array}{c} \begin{array}{cc} \cdots & R_{1\to n}\\ \cdots & R_{2\to n} \end{array}\\ \begin{array}{cc} \cdots & \vdots \\ \cdots & R_{x\to n} \end{array}\\ \begin{array}{cc} \cdots & \vdots \\ \cdots & R_{n\to n} \end{array} \end{array}\right]$$

The benefit of the transformation of 2D matrix P (see Eq. (7)) is the fast calculation by using Hahn moments calculation. The transformation matrix was done into P using a hold function discussed in recent study (Akmal, Rasool & Khan, 2017) and all moments are computed up to third-degree by using the elements of P. Two-dimension matrix used only orthogonal input, therefore we computed Hahn moments. To restore the benchmark dataset used the inverse function of orthogonal Hahn moment. By computing the Hahn moments to ‘N’ order, the following Eq. (8) was used:

(8) $$h_{n}^{u,x}\left(r,\;P\right)={\left(P+x-1\right)}_{n}{\left(P-1\right)}_{n}\;\times \overset{n}{\underset{j=0}{\sum }}{\left(-1\right)}^{j}\frac{{\left(-n\right)}_{j}{\left(-r\right)}_{j}{\left(2P+\;u+x-n-1\right)}_{j}}{{\left(P+x-1\right)}_{j}{\left(P-1\right)}_{j}}.\frac{1}{j!}$$

According to Eq. (8), the pochammer symbol and the Gamma operator are discussed in the review study (Akmal, Rasool & Khan, 2017). The following Eq. (9) is used for the computation of normalized orthogonal Hahn moments.

(9) $$H_{qr}=\;\overset{P-1}{\underset{i=0}{\sum }}\overset{P-1}{\underset{j=0}{\sum }}\gamma_{qr}\;\tilde{h_{q}^{u,x}}\left(i,P\right)\tilde{h_{r}^{u,x}}\left(j,P\right),\;\;n=0,\;1,\ldots ,\;P-1\;$$

Vital information has been stored in protein samples by calculating the central moments which are related to mean, variance, and asymmetry. The following Eq. (10) is given for the calculation of the central moment.

(10) $$H_{qr}=\;\underset{i}{\sum }\underset{j}{\sum }{\left(i-\bar{x}\right)}^{i}\;{\left(j-\bar{y}\right)}^{j}\gamma \left(i,j\right)$$

To store the essential protein samples of the dataset by calculating raw moments using the property of probability distribution as followed in the given Eq. (11):

(11) $$M_{qr}=\;\underset{i}{\sum }\underset{j}{\sum }i^{q}j^{r}\gamma \left(i,j\right)$$

The raw moments are calculated by the third degree which is r + S and M00, M01, M09, M10, M19, M20, M29, M30, and M03.

#### Frequency vector (FV)

In a benchmark dataset, one of the important parameters is frequency distribution, which gives valuable information about the distribution of the dataset for each sample of protein. Frequency is store in the form of a vector, this frequency is calculated for every amino acid, and this is called the frequency vector. The frequency vector tells us about the distribution and composition of the protein sample sequence. The computation of the frequency vector is defined in Eq. (12) as follows:

(12) $$FV=\left\{r_{1},r_{2},\ldots ,r_{21}\right\}$$

where $r_{i}$ explaining the frequency of each single amino acid residue in alphabetical order.

#### Position relative incidence matrix (PRIM)

The relative position of amino acid is directly related to the physical characteristics of the protein. The position relative incidence matrix (PRIM) is the relative position of amino acid in the polypeptide chains. The input query of protein formation is the basic step for feature extraction and the size of protein sequences builds PRIM and frequency matrix (FM). The matrix is very helpful for the computation of moments by which the feature vectors are formed. A matrix (21×21) is formed called Z_PRIM_ and it shows the protein sequences with relative associated information of residue through Eq. (13) as follow:

(13) $$Z_{PRIM}=\;\left[\begin{array}{c} \begin{array}{cc} Z_{1\to 1} & Z_{1\to 2}\\ Z_{2\to 1} & Z_{2\to 2} \end{array}\\ \begin{array}{cc} \vdots & \vdots \\ Z_{i\to 1} & Z_{i\to 2} \end{array}\\ \begin{array}{cc} \vdots & \vdots \\ Z_{N\to 1} & Z_{N\to 2} \end{array} \end{array}\begin{array}{c} \begin{array}{cc} \cdots & Z_{1\to j}\\ \cdots & Z_{2\to j} \end{array}\\ \begin{array}{cc} \cdots & \vdots \\ \cdots & Z_{i\to j} \end{array}\\ \begin{array}{cc} \cdots & \vdots \\ \cdots & Z_{N\to j} \end{array} \end{array}\begin{array}{c} \begin{array}{cc} \cdots & Z_{1\to 21}\\ \cdots & Z_{2\to 21} \end{array}\\ \begin{array}{cc} \cdots & \vdots \\ \cdots & Z_{i\to 21} \end{array}\\ \begin{array}{cc} \cdots & \vdots \\ \cdots & Z_{N\to 21} \end{array} \end{array}\right]\;\;\;\;\;\;\;\;\;\;$$

In the Z_PRIM_ matrix, element Z_ij_ is the sum value of *i*^*th*^ residue by calculating the relative position with the first happening of *j*^*th*^ residue. PRIM gives up to 441 coefficients which is a huge number, therefore computed statistical moments using PRIM as the input for the minimization of coefficient and it attains thirty coefficients.

#### Reverse position relative incidence matrix (R-PRIM)

The PRIM matrix has information regarding the relative position of amino acids along with the polypeptide chains. Sample of protein sequences with reverse input is calculated by using Z_PRIM_ for finding out the unclear hidden features from the potential sequences of protein containing the similar position uncertainty of protein. Below Z_R-PRIM_ is calculated as Eq. (14):

(14) $$Z_{R\text{-}PRIM}=\;\left[\begin{array}{c} \begin{array}{cc} Z_{1\to 1} & Z_{1\to 2}\\ Z_{2\to 1} & Z_{2\to 2} \end{array}\\ \begin{array}{cc} \vdots & \vdots \\ Z_{i\to 1} & Z_{i\to 2} \end{array}\\ \begin{array}{cc} \vdots & \vdots \\ Z_{N\to 1} & Z_{N\to 2} \end{array} \end{array}\begin{array}{c} \begin{array}{cc} \cdots & Z_{1\to j}\\ \cdots & Z_{2\to j} \end{array}\\ \begin{array}{cc} \cdots & \vdots \\ \cdots & Z_{i\to j} \end{array}\\ \begin{array}{cc} \cdots & \vdots \\ \cdots & Z_{N\to j} \end{array} \end{array}\begin{array}{c} \begin{array}{cc} \cdots & Z_{1\to 21}\\ \cdots & Z_{2\to 21} \end{array}\\ \begin{array}{cc} \cdots & \vdots \\ \cdots & Z_{i\to 21} \end{array}\\ \begin{array}{cc} \cdots & \vdots \\ \cdots & Z_{N\to 21} \end{array} \end{array}\right]$$

For decreasing the dimensionality of Z_R-PRIM_, by applying the same method to perform the coefficient reduction and statistical moments which gives back the 441 coefficients similar to Z_PRIM_, and obtained thirty coefficients.

#### Accumulative absolute position incidence vector (AAPIV)

The frequency matrix gives the information about the unclear features of samples of protein in the polypeptide chains but the accumulative absolute position incidence vector (AAPIV) gives the information of relative positional information regarding amino acid residues. Having the 21 amino acid that each single amino acid used for the sum of original values by the residues that may exist in the primary structure as Eq. (15):

(15) $$AAPIV=\left\{u_{1},u_{2},\ldots ,u_{21}\right\}$$

The computation of the arbitrary ‘$u_{i}$’ element of AAPIV as given below as Eq. (16):

(16) $$u_{i}=\overset{n}{\underset{j=1}{\sum }}P_{j}$$

#### Reverse accumulative absolute position incidence vector (R-AAPIV)

RAAPIV is formed by reversing the primary sequence of the protein and computed with the similar technique of AAPIV to know the obscure properties regarding relative positional data. Calculated the R-AAPIV by utilizing the reverse proteins sequences samples as Eq. (17):

(17) $$R-AAPIV=\left\{u_{1},u_{2},\ldots ,u_{21}\right\}$$

### Classification algorithm

**Figure 4:**
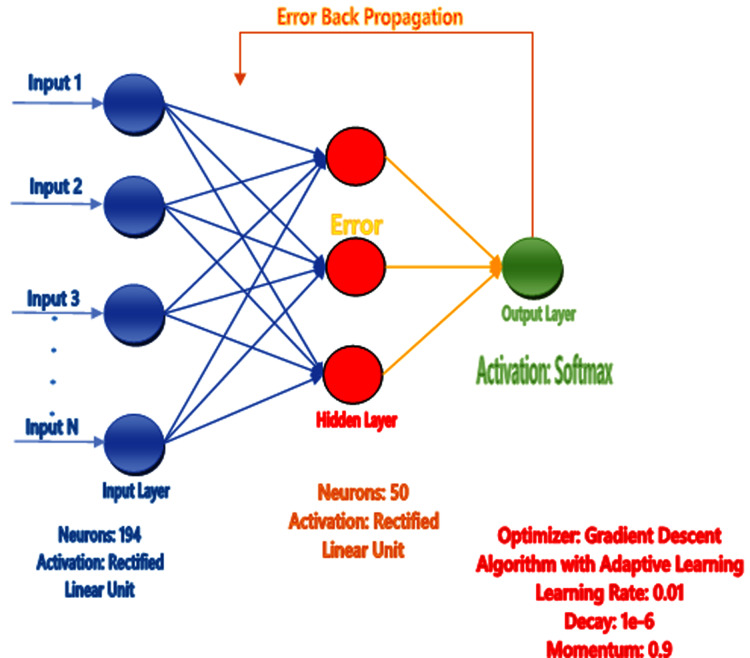
Proposed neural network prediction model.

The human brain comprises billions of neurons and they all have individual features and perform various tasks, get knowledge, take actions, and exchange information. When the neurons are operated, the Brain gains the information and performs the tasks according to conditions without having any observation or experience. The artificial neural network mechanism is constructed from the brain like the system to learn from the pattern, and past knowledge for various issues. It comprises particular neurons that perform various functions. They get the details from neurons, perform actions, and then utilize the design or model from various examples and experiences. The ANN acts on 2 models, first one is training where ANN is trained on specific data and the targeted attribute is given from the dataset to learn the pattern. The second one is testing in which we test our predicted model by giving the unseen data to its input and find the best result using trained examples and accessible information (Jiang et al., 2016) as shown in Fig. 4.

Artificial neural network (AAN) was proposed as a prediction model in our study and tuning of the model for the correction of error backpropagation was used. Extraction of feature in the form of feature vectors like Hahn, raw and central moments of sequence matrix, SVV, FV, PRIM, RPRIM, AAPIV, and RAAPIV was performed on the benchmark dataset of sample protein. Feature Vector (FV) stores the final features of the positional protein sample and the size of these features are 194.The entire feature vector (FV) was formed into the input feature matrix (IFM), where each row of IFM communicated to a single sample of protein. The output matrix (OM) was also formed for the sample output, where all the class attributes of the corresponding elements in IFM were used. Matrix IFM and OM were used for the training of ANN (See Fig. 4), in short IFM was used for getting input and OM was used for the output and compute errors during learning the model through the backpropagation technique (Jiang et al., 2016).

The scikit-learn library was used to implement the neural network. The “max_iter” parameter was increased from the default parameter value of 200 to 437. The “max_iter” parameter value was optimized to 437 using hyper parameter tuning methods and optimal value for the parameter was searched using the successive halving technique in scikit-learn. The searching space for the parameters “hidden_layer_sizes”, “learning_rate” and “momentum” was (10–50), (0.0001–0.1) and (0–1) respectively. These parameters were optimized to 23, 0.001 and 0.7 for the parameters “hidden_layer_sizes”, “learning_rate” and “momentum”, respectively, after successful halving technique. One of the key findings observed during the experimentation process was that “max_iter” with more than 500 iterations minimally contributed to the accuracy of the classifier substantially.

## Results

Evaluation of the new machine learning prediction model is the essential step for the success rate and completeness of the model (Chou, 2011). For the best examination of the model, we must focus on two parts of the research study of the prediction model, the type of metrics we can use to represent the model prediction standard and the test methods that are best for scoring the metrics.

### Evaluation metrics

For the correctness and the efficiency of the proposed model, we must consider four main points’ metrics: (1) Find out the accuracy Acc of the proposed method, (2) Find out sensitivity Sn of the prediction model, (3) Find out specificity Sp of a predictor, and (4) Find out MCC for the strength of a predictor. The traditional metrics are mostly used in math to validate the accuracy of the prediction model, but it may be a very complex job for the biologist to perceive and use these metrics. For this reason, Mathew’s correlation coefficient (MCC) is the best matrices used for the prediction model reliability and strength. Most of the symbols used in protein signal peptide by Chou (Chou), a collection of four built-in equations were worked (Chou, 2001b, 2015; Xu et al., 2013; Arif, Hayat & Jan, 2018) as follow in Eq. (18).

(18) $$\left\{ \begin{array}{c} Sn=1‒\frac{{ℵ}_{‒}^{+}}{{ℵ}^{+}}\\ sp=1‒\frac{{ℵ}_{+}^{-}}{{ℵ}^{-}}\\ Accuracy=1‒\frac{{ℵ}_{‒}^{+}+{ℵ}_{+}^{-}}{{ℵ}^{+}+{ℵ}^{-}}\\ MCC=\frac{1‒\left(\frac{{ℵ}_{‒}^{+}}{{ℵ}^{+}}+\frac{{ℵ}_{+}^{-}}{{ℵ}^{-}}\right)}{\sqrt{\left(1+\frac{{ℵ}_{+}^{-}-{ℵ}_{-}^{+}}{{ℵ}^{+}}\right)\left(1+\frac{{ℵ}_{-}^{+}-{ℵ}_{+}^{-}}{{ℵ}^{-}}\right)}} \end{array}\right.$$

As explained in above Eq. (18) that when the value of ${ℵ}_{-}^{+}=0$ which simply means that the predictor predicts all the SUMOk sites correctly and no one reaming to predict the sites, therefore we have the sensitivity Sn = 1. On the other side if ${ℵ}_{-}^{+}={ℵ}^{+}$ which means the predictor predicts all the SUMOk sites incorrectly, therefore we have the sensitivity Sn = 0. Similarly, when ${ℵ}_{+}^{-}=0$ which means the predictor predicts all the non-SUMOk sites correctly and no one reaming to predict, therefore we get specificity Sp = 1. Further, if we have ${ℵ}_{+}^{-}={ℵ}^{-}$ which means that the predictor predicts all the non-SUMOk sites incorrectly, therefore we get the specificity Sp = 0. Furthermore, when ${ℵ}_{-}^{+}={ℵ}_{+}^{-}$ which means that the predictor predicts correctly for all positive dataset as well as for all negative dataset and no remaining sites for prediction, that gives the overall accuracy Acc = 0 and MCC = 1. On the other hand, if ${ℵ}_{-}^{+}={ℵ}^{+}$ and ${ℵ}_{+}^{-}={ℵ}^{-}$ which means that the predictor predict all the values for the positive dataset and negative dataset incorrectly and we get MCC = −1 and Acc = 0; whereas if ${ℵ}_{-}^{+}=\frac{{ℵ}^{+}}{2}$ and ${ℵ}_{+}^{-}=\frac{{ℵ}^{-}}{2}$ it gives the MCC = 0 and Acc = 0.5, which means nothing, and a random guess is better. Therefore Eq. (18) provides the detailed meaning of stability, comprehensive accuracy, specificity, and sensitivity for a better understanding, as discussed by many investigators (Chen et al., 2016b; Xiao et al., 2016).

In recent publications (Lin et al., 2014; Xu et al., 2014; Chen et al., 2016c; Zhang et al., 2016; Ehsan et al., 2018), these set of metrics were utilized for research in state-of-the-art methods. According to Eq. (18), SUMOk sites or non-SUMOk sites are applicable only for the binary classification data. Multi-label class problem is not applicable for this kind of solution, because this problem is different in biomedicine (Xiao et al., 2013) and biological (Xiao, Wu & Chou, 2011; Chou, Wu & Xiao, 2012; Lin et al., 2013), therefore a different set of metrics required for this problem as explained in this study (Chou, 2013).

#### Self-consistency tests

Self-consistency testing was implemented for iSUMOk-PseAAC sites, which mean we have to train and test on the benchmark dataset (Chou, 2011). It is mostly used when the results are already known and saved in dataset; here we used true positive results for validation of our model. Results of our validation are shown in Table 1, which show all the predicted and actual classification. This tells us about the overall performance of our model.

**Table 1:** Self-consistency tests for sumoylation sites.

Predictor	Evaluation metrics				
	Acc (%)	Sp (%)	Sn (%)	MCC (%)	AUCs
SVM	71.62	72.54	70.77	0.43282	0.78
KNN	79.14	82.15	76.64	0.58543	0.87
Neural Network	100	100	100	1.0	1.0

#### Independent tests

Independent dataset testing plays a very important role in the evaluation of machine learning models because this testing dataset is divided into two parts one is the training dataset and the other is the testing dataset. The dividing ratio of the dataset can be different. In this research 70:30 ratio is used for training and testing and measure the highest accuracy which is given below in Table 2.

**Table 2:** Independent tests for sumoylation sites.

Predictor	Acc (%)	Sp (%)	Sn (%)	MCC (%)	ACC
SVM	70.44	71.84	69.18	0.40959	0.67
KNN	65.97	68.67	63.9	0.32277	0.56
iSUMOk-PseACC (Neural Network)	88.60	89.29	88.16	0.7651	0.94

#### Receiver operating characteristics (ROC)

**Figure 5:**
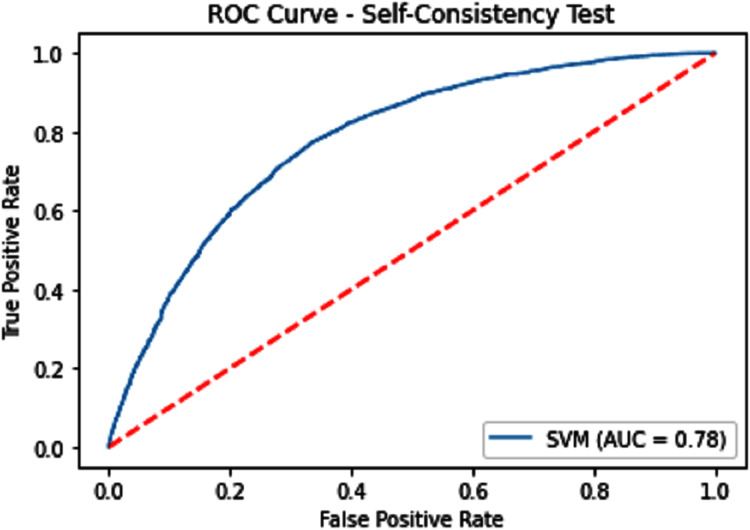
Self-consistency test ROC for SVM.

**Figure 6:**
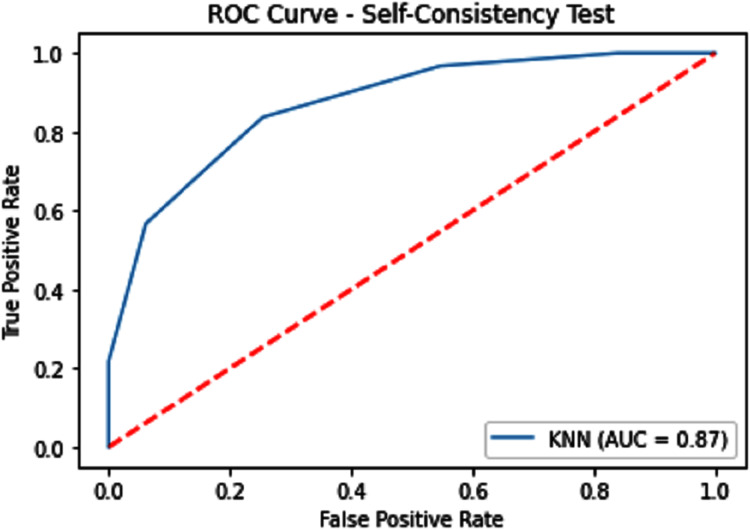
Self-consistency test ROC for KNN.

**Figure 7:**
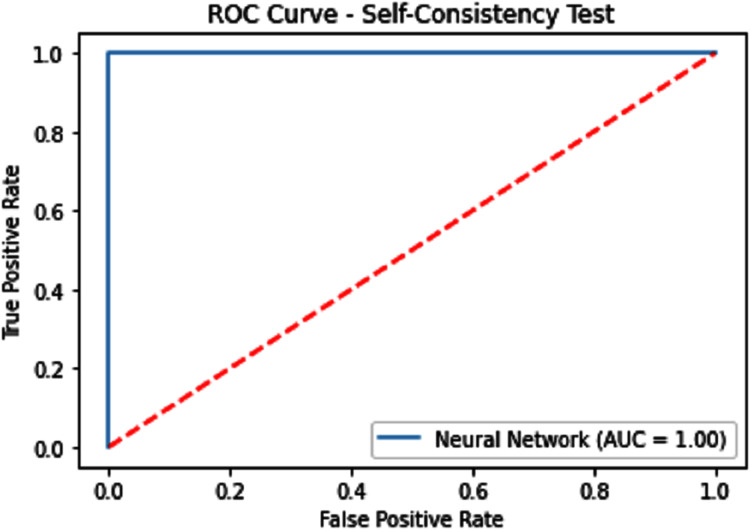
Self-consistency test ROC for neural network.

**Figure 8:**
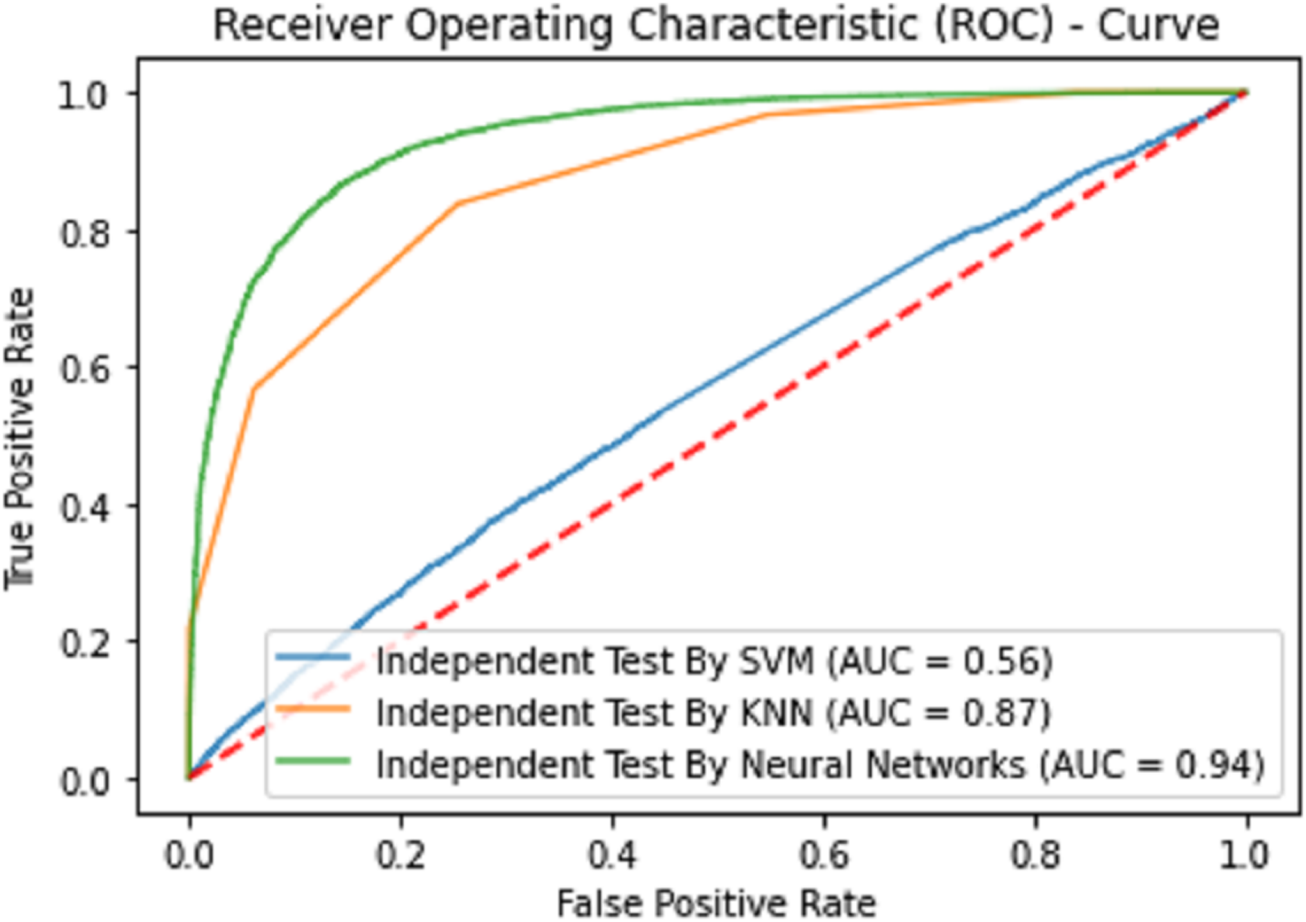
Independent test ROCs for Sumoylation sites.

The performance measure is the important step of every machine learning model, therefore we draw Area under the curve receiver operating characteristic (AUC-ROC) for classification problem. It represents the model’s accuracy in the form of true positive rate and false-positive rate. If the AUC is higher or near to 1 which means the model performed well and separate the class correctly. If the AUC is worst or near to 0 which means the performance of the model is poor and fails to separate the class. If AUC is 0.5, it means the performance of the model is neutral. This curve is plotted with TPR against the FPR where TPR is on the *y*-axis and FPR is on the *x*-axis. The ROCs for Self Consistency Tests are shown in Figs. 5–7. The ROCs for Independent Tests are shown in Fig. 8.

#### 10-Fold cross-validation test

**Figure 9:**
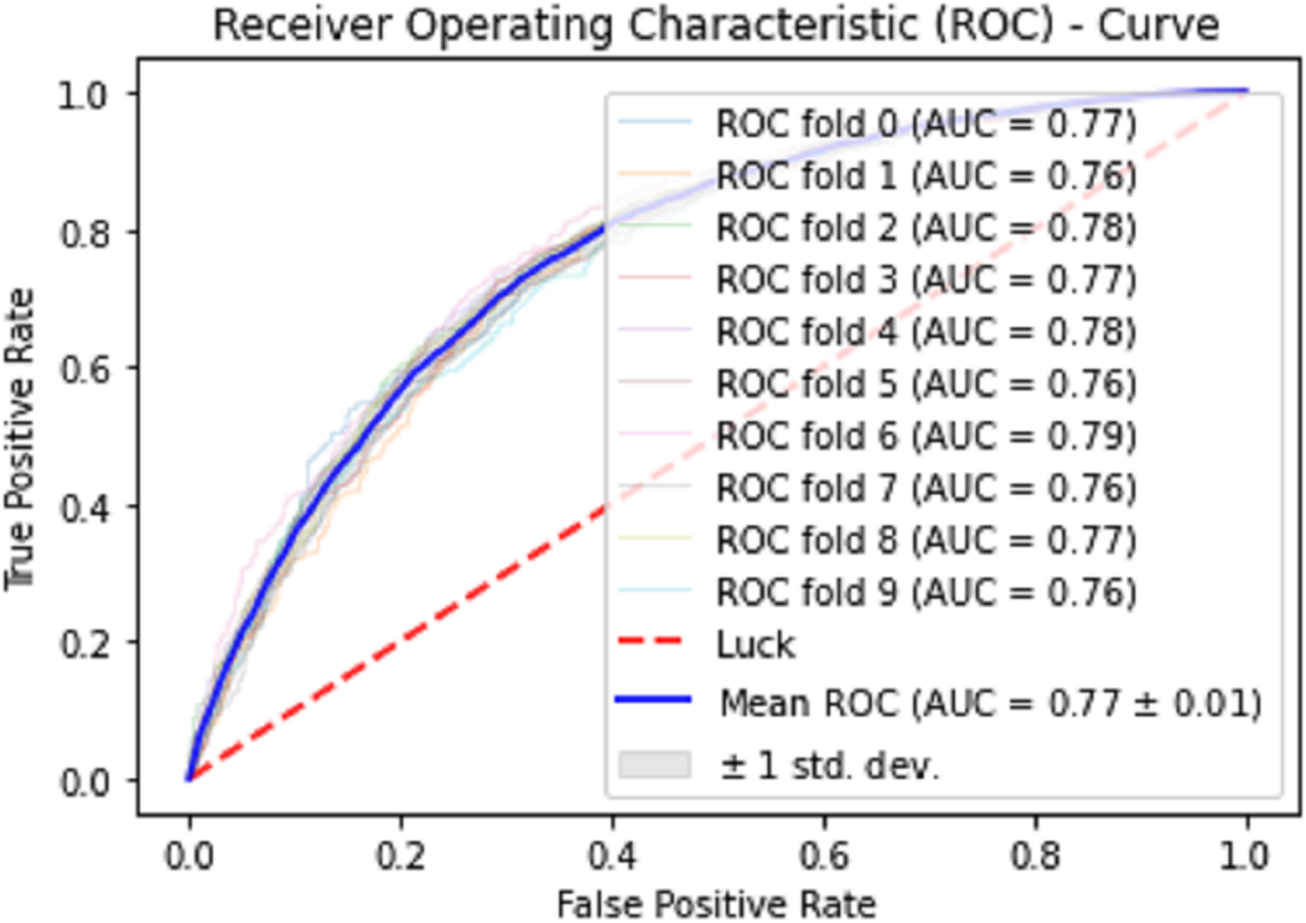
10-Fold cross-validation test ROCs (SVM) for sumoylation sites.

**Figure 10:**
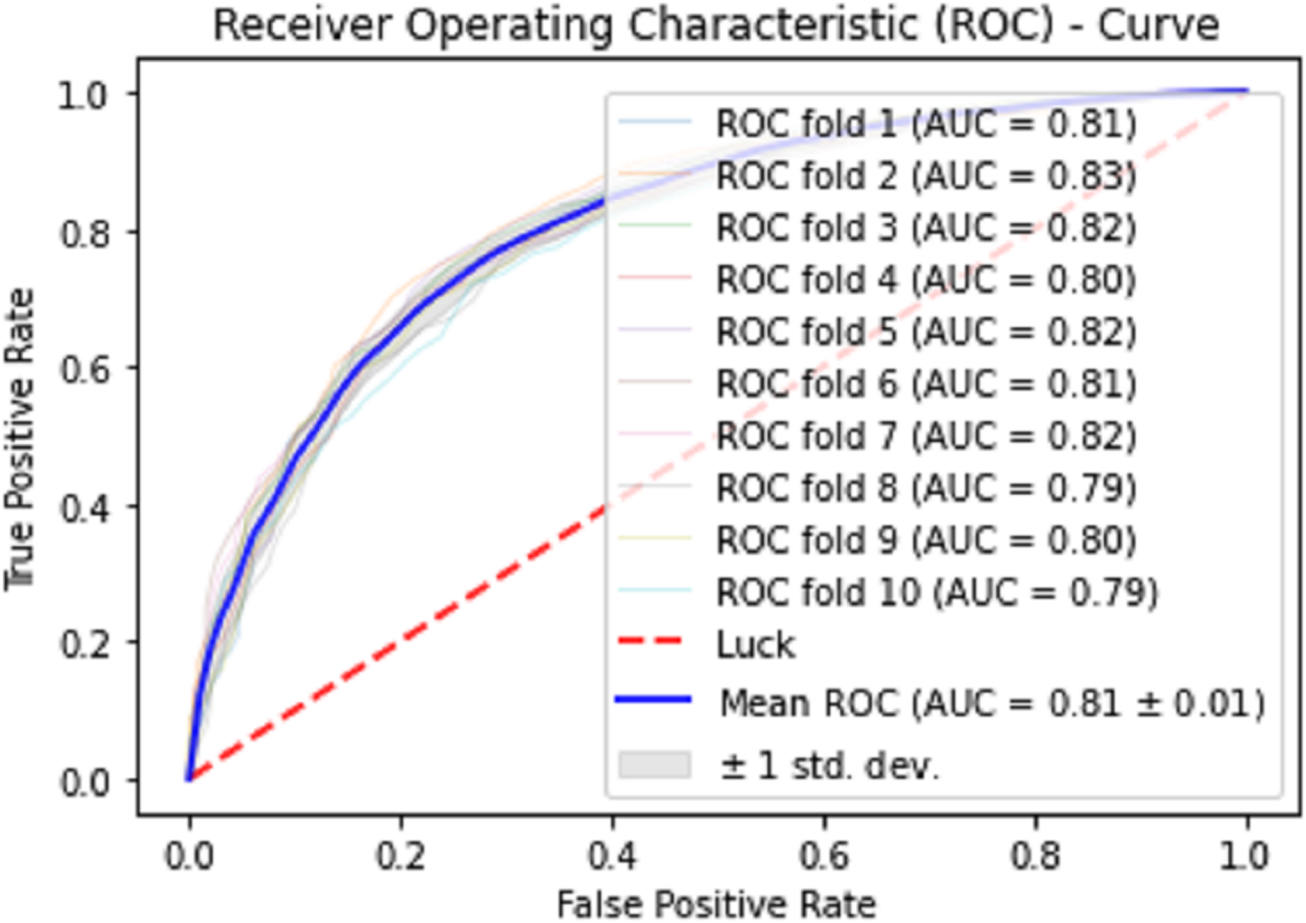
10-Fold cross-validation test ROCs (KNN) for sumoylation sites.

**Figure 11:**
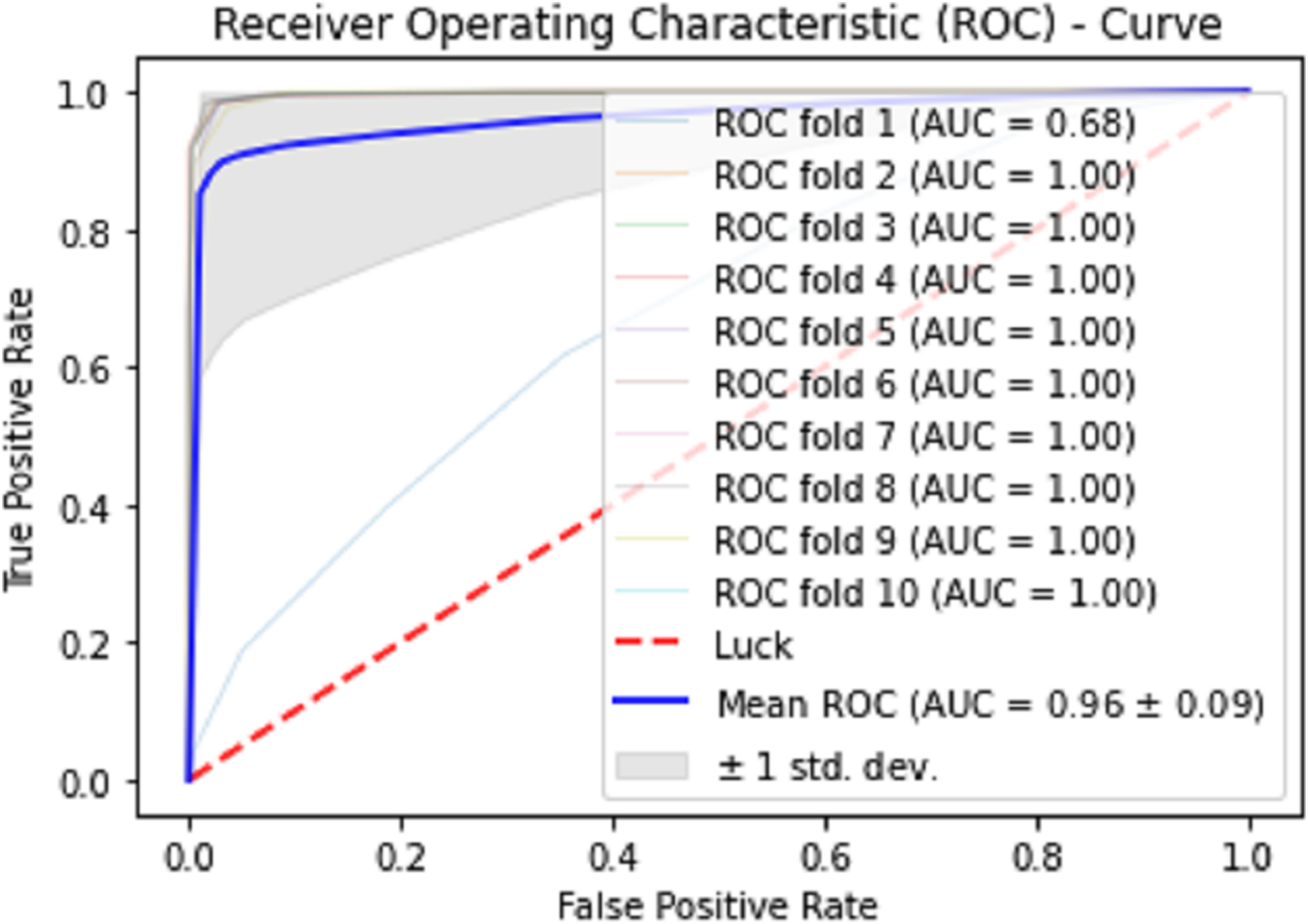
10-Fold cross-validation test ROCs (neural networks) for sumoylation sites.

Normally a dataset for prediction model is needed which should be labeled, in some cases which are not easy to obtain, so this testing of the dataset is taken from the original dataset, which was also used for training, this set is very useful to test the performance of the model. Especially in cross-validation, a dataset is divided into K parts where K can be any number and then one part of that data is kept for testing while other parts are used for training purposes. Next, a new part of data is chosen as the testing sample while others are used for training, this is repeated until all parts are tested. Finally, the average of all the results is calculated. In our case, the value of K = 10 and the result was average of all the accuracy for each fold. Our results are illustrated in Table 3. Figures 9–11 show the 10 Fold cross validation results ROCs.

**Table 3:** 10-Fold cross-validation tests for sumoylation sites.

Predictor	Accuracy Metrics			
	Acc (%)	Sp (%)	Sn (%)	MCC (%)
SVM	70.70	68.90	72.50	0.4144
KNN	73.69	74.90	72.48	0.4743
iSUMOk-PseAAC	94.51	94.24	94.79	0.8903

## Discussions

There have been many predictors to predict the Sumoylation site, but their results not good as compared to our iSUMOk-PseAAC predictor. Recently Chang et al. (2018) proposed the SUMOgo predictor, which shows the highest accuracy of other Sumoylation site prediction tools through independent testing. To detect the overall accuracy of a prediction model, the dataset consisted of 867 positive sets and 18,824 negative sets collected from three different databases like for training purposes dataset collected from UniProtKB and for testing collected from dbPTM and PhosphoSitePlus. The same dataset was used in this study by performing an independent dataset testing of iSUMOkPseAAC and its contrast with SUMOgo (Chang et al., 2018), SUMOsp2.0 (Xue et al., 2006), JASSA (Beauclair et al., 2015), GPS-SUMO (Zhao et al., 2014), and PCI-SUMO (Green, Dmochowski & Golshani, 2006).

**Figure 12:**
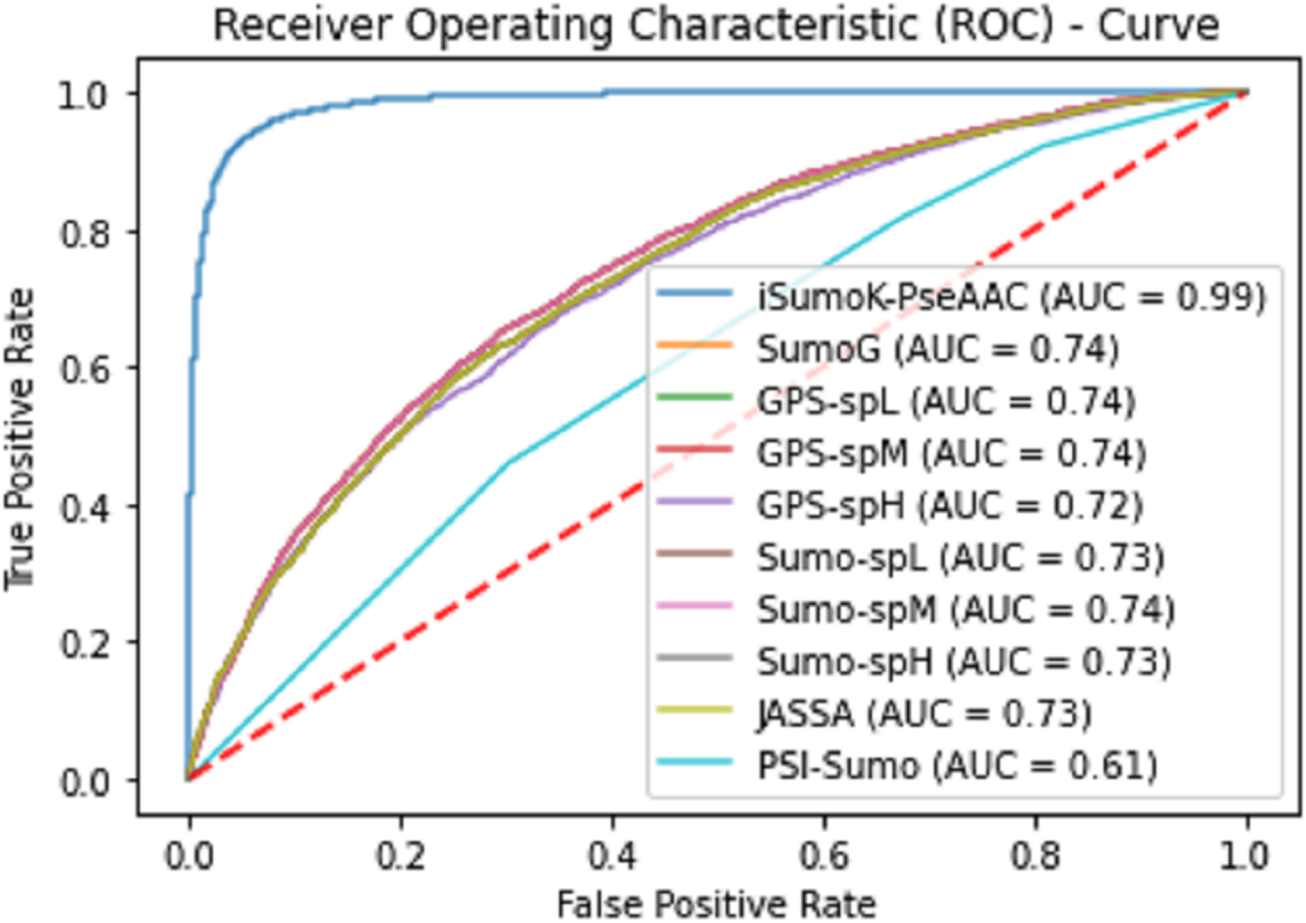
Comparative analysis ROCs of state-of-the-art methods.

The overall accuracy in terms of ACC, Sp, Sn, and MCC is much higher in the proposed Sumoylation tool because iSumok-PseAAC performed well under statistical moments based features extractions as shown in Table 4 of their accuracy metrics. The PseAAC methods were utilized by the position relative features and statistical calculation for prediction of sumoylation sites. First of all 20 residues of the amino acid are surrounding left and 20 rights for targeted residue, after that computed statistical moments and for reduction of dimensionality constructed the frequency vector, site vicinity vector, PRIM, RPRIM, AAPIV, and RAAPIV. Finally, the composition of sequence and position relative feature is given to predictor for prediction. The Fig. 12 represents the ROCs analysis for state-of-the-art methods.

**Table 4:** Performance of state-of-the-art methods in sumoylation site predictions.

State-of-the-art	Sn	Sp	ACC	Precision	MCC
iSumok-PseAAC	0.9451	0.9424	0.9479	0.9714	0.8903
Sumogo[16]	0.592	0.896	0.744	0.850	0.511
C-iSumo [109]	0.734	0.757	0.746	–	0.494
GPS-L[1]	0.668	0.810	0.739	0.778	0.482
GPS-M[1]	0.642	0.833	0.738	0.794	0.484
GPS-H[1]	0.540	0.897	0.719	0.840	0.468
SUMOsp2.0_L[13]	0.709	0.750	0.730	0.739	0.460
SUMOsp2.0_M[13]	0.655	0.823	0.739	0.787	0.485
SUMOsp2.0_H[13]	0.608	0.873	0.740	0.827	0.498
JASSA[2]	0.654	0.808	0.731	0.773	0.467
PCI-SUMO[5]	0.687	0.530	0.609	0.594	0.220

## Web-server development

The final step of Chou’s five-step rule (Chou, 2011) is the Web Server, which plays an important role in various computational analyses and findings. In recent research studies (Cheng et al., 2018; Cheng, Xiao & Chou, 2018c; Chou, Cheng & Xiao, 2018; Xiao et al., 2018), the effect of computational biology on medical science as effectively enhanced the availability, and also this server provides user-friendly environment (Chou & Shen, 2009) for the easiness of user as well as biologists, which drives the medical science (Chou, 2015) into an exceptional revolution (Chou, 2017). The software code for the current study is provided at GitHub: https://github.com/csbioinfopk/iSumoK-PseAAC. The webserver to the current study will be provided for the research community in near future.

## Conclusions

The presence of SUMO modification has occurred for over 10 years. Sumoylation plays an essential role in the regulation of various cellular functions. Sumoylation progress towards various biological processes, diseases, and medication; therefore, it is considered one of the significant aspects of cellular functions along with transcriptional ordinance, protein reliability, and the development throughout the cell cycle. Sumoylation relations with many different types of diseases like cancer, diabetes, inherit heart flaws, and most important neurodegenerative diseases which are directly linked to Sumoylation synchronization and modulation. Consequently, the classification of potential Sumoylation sites is beneficial, for this purpose we propose a SUMOk prediction tool, which is more accurate and efficient for easy experimental results followed by the five-step rule. To test the accuracy of iSUMOk-PseAAC, 10-fold cross-validation was implemented with the help of metrics. We achieved the result of 10 fold cross-validation with 94.51% accuracy, 94.24% sensitivity, 94.79% specificity, and 0.8903% MCC. For that reason, iSUMOk-PseAAC predictors are very helpful for predicting Sumoylation sites in an accurate and precise manner, although the results of the proposed model would be better by grouping the growing number of SUMO sites in a sequence of the protein.

## Supplemental Information

10.7717/peerj.11581/supp-1 Supplemental Information 1 Negative Sequences.

10.7717/peerj.11581/supp-2 Supplemental Information 2 Positive Sequences.

10.7717/peerj.11581/supp-3 Supplemental Information 3 Negative Sequences for Training.

10.7717/peerj.11581/supp-4 Supplemental Information 4 Positive Sequences for Training.

10.7717/peerj.11581/supp-5 Supplemental Information 5 Negative Sequences for Independent Testing.

10.7717/peerj.11581/supp-6 Supplemental Information 6 Positive Sequences for Independent Testing.

## Additional Information and Declarations

### Data Availability

The following information was supplied regarding data availability: Data and code are available at GitHub: https://github.com/csbioinfopk/iSumoK-PseAAC.
